# Pennsylvania Hospital

**Published:** 1849-06

**Authors:** Spencer Sergeant

**Affiliations:** Resident Physician; Pennsylvania Hospital


PENNSYLVANIA HOSPITAL.—*Surgical Wards.—Service of* DR. PEACE.

*Cases discharged since April* 15*th*, 1849.

| | Cured. | By request. | Died. |
|---|---|---|---|
| Abscess, | 1 | 0 | 0 |
| Amputation, | 1 | 0 | 0 |
| Amaurosis, | 1 | 0 | 0 |
| Burn, | 1 | 0 | 0 |
| Cancer of lip, | 1 | 0 | 0 |
| Caries, | 1 | 0 | 0 |
| Concussion of Brain, | 2 | 0 | 0 |
| Contusions, | 5 | 0 | 0 |
| Deformity, | 1 | 1 | 0 |
| Enlarged testicle, | 0 | 1 | 0 |
| Erythema, | 1 | 0 | 0 |
| Fistula in ano, | 0 | 1 | 0 |
| " recto-vaginal. | 0 | 1 | 0 |
| Fractures, simple 7, viz.: | | | |
| Lower jaw, | 1 | 0 | 0 |
| Clavicle, | 1 | 0 | 0 |
| Ribs, | 1 | 0 | 0 |
| Arm, | 2 | 0 | 0 |
| Arm and fore-arm. | 1 | 0 | 0 |
| Fractures, compound 4, viz.: | | | |
| Skull, | 1 | 0 | 0 |
| Hand, | 1 | 0 | 0 |
| Fore-arm, | 1 | 0 | 0 |
| Leg, | 1 | 0 | 0 |
| Frost-bite, | 1 | 0 | 0 |
| Inflamed knee-joint, | 1 | 0 | 0 |
| " testicle, | 1 | 0 | 0 |
| Necrosis, | 1 | 0 | 0 |
| Paronychia, | 2 | 0 | 0 |
| Syphilis, | 5 | 0 | 1 |
| Stricture of urethra, | 1 | 0 | 0 |
| Ulcers, | 6 | 0 | 0 |
| Wounds 5, viz.: | | | |
| Incised, | 3 | 0 | 0 |
| Lacerated, | 1 | 0 | 0 |
| Gun shot, | 1 | 0 | 0 |
| | 48 | 4 | 1 |

The case of compound fracture of the leg is of some interest, from the fact that the patient refused to submit to amputation which was recommended by the surgeons. With several chances against him he drew the one in his favour. Similar cases are of not unfrequent occurrence in every large hospital, and cannot in any way influence the general rules to be followed with like injuries.

John Doran, aged 23, a healthy looking young Irishman, was admitted the evening of November 8th, 1848, with a compound fracture of the left leg, about the middle, caused by the caving in of a bank of earth. The soft parts were extensively lacerated and contused, and about three inches of the upper fragment of the tibia projected through the wound, but the main arteries of the limb were untouched. On examining the limb more attentively, an old cicatrix with depressions here and there was discovered, covering nearly the whole of the shin, and on questioning the patient he stated that several years before he had had disease of the bones of that leg, and that several pieces had come away.

In consideration of the extent of the recent injury, and of the probability of the sloughing which would almost inevitably attack the contused and lacerated soft parts involving the old cicatrix, and so exposing more of the bone, amputation was recommended by the surgeons, but refused by the patient.

The accident had happened about four hours before admission, and reaction was fully established. Pulse about 85, moderately full.

The limb was placed in a fracture box and dressed with lint wet with cold water.

The next day he was found to have passed a restless night, but the constitutional disturbance was very slight. Pulse 85. Limb swollen and painful.

As soon as suppuration was established, the limb was placed in bran, and the sixth and eighth days small sloughs separated from the upper part of the wounds. No constitutional irritation whatever ; pulse of ordinary force and frequency ; appetite good.

The exposed portion of the tibia became necrosed, separated, and was removed at the expiration of the fourth week, and by the first of January the wounds had nearly cicatrized. Union, however, was not complete for some time afterwards, but he was allowed to arise the latter part of February, and was discharged well April 16th, 1849.

The amount of constitutional irritation which follows injuries, varies very much in different individuals. I have before* related a case in which intense constitutional disorder, terminating fatally, was the result of a lacerated wound of the little finger. In the following case a bone was broken, and a large joint extensively opened, and still the natural tranquillity of the system was but slightly disturbed.

John Malone, Irish, aged 22, was admitted March 31st, 1849, with a compound fracture of the right patella, caused by a kick from a horse, two hours before.

The patella was broken transversely at the lower part; and through two external wounds, each about an inch in length, the finger could be passed deeply into the joint, which was distended with effused blood.

He did not complain of much pain. Pulse 86, and moderately full.

The wounds were closed with adhesive plaster, and lint dipped in the white of egg, and the limb was confined on a straight splint and, both to relax the extensor muscles and favour the return of blood, elevated on an inclined plane. Ordered a calomel purge followed by salts, and at night an opiate. Cold water to the knee and low diet.

The next day the knee was very much swollen, red and painful, but the constitutional symptoms were but trifling. Pulse 86. Skin moderately warm. Ordered a diaphoretic and sedative mixture.

*April* 2*d*. Knee about the same. Pulse 84. Sleeps well.

5*th*. Knee still much swollen and red, with some signs of suppuration. Removed the cold water dressing and applied a poultice. Pulse 84.

Discontinue the diaphoretic mixture.

14*th*. Knee much reduced in size, suppurating freely. General symptoms have continued without change.

23*d*. Suppuration much less. Knee very nearly of its natural size.

*May* 1*st*. Wounds entirely cicatrized, ligamentous union of less than half an inch.

15*th*. Union firmer than in many simple fractures of the patella

* Examiner, March, 1849.

of eight weeks standing. He is now regaining the motions of the joint and almost fit to be discharged.

A sailor aged 43 was admitted into the Hospital April 12th, 1849, with secondary syphilis. He was said to have had syphilis several times, and each time to have been treated with mercury. At the time of his admission in addition to pains of which he complained most, he had a papular eruption on the skin and considerable irritation about the upper part of the throat. He was placed on the use of iodide of potassium, and an astringent wash for his throat.

He remained without much change until May 2d, when he was seized with a sudden dyspnœa, and difficulty of expectoration. There was no alteration of the voice, but the respiration was audible at a short distance and prolonged. Very slight pain was occasioned by pressure on the larynx. Examination of the chest by auscultation detected no abnormal sounds. He was relieved by the use of warm baths, blisters to the throat, and a nauseating solution.

On the 4th he had another attack, which was relieved by the same remedies.

During the fifth he was much improved, but on the morning of the 6th he had an attack so sudden and severe as to cause his death in a few moments.

The autopsy was made twenty-four hours after death. The trachea and larynx with the tongue were removed from the body, and on slitting up the larynx the posterior part of the cricoid and a part of the thyroid cartilage were found converted into bone, carious, and containing a loose sequestrum, exhaling a most fetid odour. The sub-mucous tissue above and to the left side of the rima glottidis was highly œdematous, and completely overhung this aperture. The lungs were congested, but otherwise they, as well as the other viscera, were healthy. Further examination detected a cartilaginous stricture at the membranous portion of the urethra.

SPENCER SERGEANT,
Resident Physician.

*Pennsylvania Hospital, May 15th*, 1849.

